# Unique LCR variations among lineages of HPV16, 18 and 45 isolates from women with normal cervical cytology in Ghana

**DOI:** 10.1186/s12985-017-0755-z

**Published:** 2017-04-21

**Authors:** Adolf K. Awua, Richard M. K. Adanu, Edwin K. Wiredu, Edwin A. Afari, Vanessa A. Zubuch, Richard H. Asmah, Alberto Severini

**Affiliations:** 1 Department of Epidemiology and Disease Control, School of Public Health, University of Ghana, Accra, Ghana; 2 Cellular and Clinical Research Centre, Radiological and Medical Sciences Research Institute, Ghana Atomic Energy Commission, Accra, Ghana; 3 Population, Family and Reproductive Health, School of Public Health, University of Ghana, Accra, Ghana; 4 Department of Pathology, School of Biomedical and Allied Health Science, University of Ghana, Korle-Bu, Accra, Ghana; 5 National Microbiology Laboratory, Public Health Agency of Canada, Winnipeg, MB Canada; 6 Department of Medical Laboratory Sciences, School of Biomedical and Allied Health Science, University of Ghana, Korle-Bu, Accra, Ghana; 7 University of Manitoba, Winnipeg, MB Canada

**Keywords:** HPV Lineage, HPV Variant, Human Papillomavirus, Long Control Region, African Lineage, Ghana

## Abstract

**Background:**

In addition to being useful for classification, sequence variations of human Papillomavirus (HPV) genotypes have been implicated in differential oncogenic potential and a differential association with the different histological forms of invasive cervical cancer. These associations have also been indicated for HPV genotype lineages and sub-lineages. In order to better understand the potential implications of lineage variation in the occurrence of cervical cancers in Ghana, we studied the lineages of the three most prevalent HPV genotypes among women with normal cytology as baseline to further studies.

**Methods:**

Of previously collected self- and health personnel-collected cervical specimen, 54, which were positive for HPV16, 18 and 45, were selected and the long control region (LCR) of each HPV genotype was separately amplified by a nested PCR. DNA sequences of 41 isolates obtained with the forward and reverse primers by Sanger sequencing were analysed.

**Results:**

Nucleotide sequence variations of the HPV16 genotypes were observed at 30 positions within the LCR (7460 – 7840). Of these, 19 were the known variations for the lineages B and C (African lineages), while the other 11 positions had variations unique to the HPV16 isolates of this study. For the HPV18 isolates, the variations were at 35 positions, 22 of which were known variations of Africa lineages and the other 13 were unique variations observed for the isolates obtained in this study (at positions 7799 and 7813). HPV45 isolates had variations at 35 positions and 2 (positions 7114 and 97) were unique to the isolates of this study.

**Conclusion:**

This study provides the first data on the lineages of HPV 16, 18 and 45 isolates from Ghana. Although the study did not obtain full genome sequence data for a comprehensive comparison with known lineages, these genotypes were predominately of the Africa lineages and had some unique sequence variations at positions that suggest potential oncogenic implications. These data will be useful for comparison with lineages of these genotypes from women with cervical lesion and all the forms of invasive cervical cancers.

**Electronic supplementary material:**

The online version of this article (doi:10.1186/s12985-017-0755-z) contains supplementary material, which is available to authorized users.

## Background

With an estimated 3052 new cases of cervical cancer in Ghana in the year 2012, cervical cancer is one of the two leading cause of cancer incidence and mortality in Ghana and in most sub-Saharan African countries. Cervical cancer is a significant health concern in Ghana in light of the facts that in spite of its high burden, cervical cancer screening programmes are still lacking. In the absence of empirical cancer registry based data, the estimated age-standardized cervical cancer incidence rate for Ghana (35.4 per 100,000) indicates a possible difference in the cervical cancer burden among close neighbours; Benin (27.6 per 100,000), Togo (21.5 per 100,000), Burkina Faso (23.3 per 100,000) and Cote d’Ivoire (21.7 per 100,000) [1].

These geographical variations in the incidence of cervical cancer are also seen in the prevalence of its etiological agent, which is the human Papillomavirus (HPV). In spite of these variations, HPV genotypes 16, 18 and 45 together are responsible for more than 80% of cervical cancer globally [2–4]. Molecular studies of each of these genotypes have shown sequence variations in some genes that have been the basis for their classification into phylogenetic linages and sub-lineages [5]. Conventionally, if the DNA sequence of the L1 gene of two HPV isolates defer by between 1 and 10%, then these two HPV isolates are variants (lineage) of a genotype. A difference of between 0.5 and 1% makes the HPVs sub-lineage (sub-variant) of a lineage [6, 7]. Furthermore, the level of nucleotide sequence identities or differences in other parts of the HPV genome (including the LCR, E6 and E7) and mostly in the full genome sequence have resulted in similar classification [5–11].

Specifically, variants of HPV16 have been classified most recently, as 4 phylogenetic lineages based on LCR and E6 open reading frames of its genome. These are, a) lineage A (also known as European-Asian, EAS lineage), consisting of at least the sub-lineages A1, A2, A3, (known previously as European, E lineage) and A4 (previously known as Asian, As lineage); b) Lineage B (known previously as African-1 (Afr-1) lineage), consisting of at least two sub-lineages B1 (Afr-1a) and B2 (Afr-1b); c) Lineage C (known previously as African-2 (Afr-2) lineage), previously consisting of the sub-lineages Afr-2a and Afr-2b, and d) lineage D (Asian-American/North American, AA/NA lineage), consisting of at least the sub-lineages, D1 (known previously as North-American (NA) lineage), D2 (known previously as Asian-American-1 (AA1) lineage), and D3 (Asian-American-2 (AA2) lineage). These groups of lineages have more than 95% sequence homology in the long control region (LCR) [5, 7, 12–14].

For HPV18 variants, two phylogenetic lineages (previously known as non-African and African) were identified based on full genome sequence analysis in an older classification. The non-African lineages had two sub-lineages namely, European (E) and Asian-American (AA) while the African lineage also had two sub-lineages, namely African 1 (Afr-1) and African-2 (Afr-2) [15]. However, with a more recent classification, three lineages have been identified; the previous non-Africa lineage was identified and designated as lineage A with five sub-lineages, designated A1 to A5. Part of the previous African lineage was identified as a separate lineage and designated lineage B with three sub-lineages, designated B1 to B3. The rest of the previous African lineage were identified as a new lineage and designated lineage C [6, 10, 15]. Also based on full genome sequence analysis, HPV 45 variants have been classified into two phylogenetic lineages, namely A and B. while lineage A has at least three sub-linages; A1, A2 and A3, lineage B has two sub-lineages, B1 and B2 [6, 8, 15].

The long control region (LCR) of the HPV genome is a 850 bp regulatory region that includes a section for regulating the transcription of HPV genes through interactions with many cellular and viral factors [13]. Therefore, sequence variation in the LCR in addition to being useful for classification has been implicated in the different extent of persistence and progression and oncogenic potential of HPV variants [15, 16]. It has also been shown that HPV genotypes (which vary in their LCR sequences) are differentially associated with different histological forms (squamous cell carcinoma (SCC); adenocarcinoma (ADC) and adenosquamous carcinoma (ADSC)) of invasive cervical cancer [17–22]. Specifically for invasive cervical cancer (ICC) reported in Ghana, HPV16 and closely related HPV types such HPV31 were common in SCC, while HPV18 and closely related types such HPV45 and HPV59 were common in ADC and ADSC [23, 24]. Such differential specificities have also been indicated for HPV variants/lineages [5, 14, 25–29].

It is therefore important for our population and in order to enhance cervical cancer screening, to study the variants of the reported most prevalent HPV genotypes in Ghana, to better understand the potential of developing cervical cancers. Although there are limited empirical data on the prevalence of human Papillomavirus (HPV) in the general population, the most prevalent HPV genotypes in Ghana are HPV 16, 18 59, and 45. Few hospital based studies on HPV and its distribution in cancers have reported, for instance, a 10% HPV prevalence among 75 women screened for cervical cancer in a tertiary hospital [30]. Three other studies, using formalin-fixed paraffin embedded (FFPE) cervical tissue samples of women diagnosed with high grade cervical lesion or invasive cervical cancer (at the same hospital) reported HPV prevalence of 93.9% [31], 98.0% [32] and 89.8% [23, 24]. Overall, only two of these studies reported the genotype specific prevalence, which indicated that HPV 18, 59, 16 and 45 were among the common genotypes in Ghana [23, 32]. However, there are no data on the variants of these common HPV genotypes in Ghana. With increasing evidence of HPV genotype, subtype and lineage specificity and association with different forms of invasive cervical cancer, subtype and lineage determination may become important in risk assessment. Additionally, kits development may depend on sequence data of HPV variants for specific targeting, particularly for the HPV 16, 18 and 45. Therefore, our study, focused on the determination of the lineages of HPV 16, 18 and 45 isolated from women participating in a community-based cervical cancer screening activity, all of whom were negative by Pap smear testing (normal cytology results).

## Methods

### Cervical specimen

Previously collected cervical specimen of women with normal cytology results, during a community based cervical cancer screening programme, were used for this study. The specimen positive for HPV16, 18 and 45 (either as a single infection or in a multiple infection) were selected and the long control region (LCR) of each HPV was separately amplified by a nested PCR to confirm the genotype present in the specimen. A total of 20 HPV16 positive specimens, 15 HPV18 positive specimens and 18 HPV45 positive specimens were used.

### DNA extraction

DNA was extracted from these specimen on a MagNA Pure LC automated system (Roche Molecular Systems, Inc, Pleasanton, USA) using 200 μL of the cell suspension and the MagNA Pure DNA Extraction Kit (Roche Molecular Systems, Inc, Pleasanton, USA) according to the manufactures specification with a modification of an addition of 15 μL of RNase to the lysis buffer for each specimen. The negative controls included for each set of extraction were PBS, DNase/RNase free water, and DNAGard, while the positive control was a suspension of SiHa and HeLa cells with an integrated HPV16 and HPV18 genome respectively. The specimen/DNA quality was assessed by the amplification of the human RNase H gene in 10 μL of DNA extract and analysed with Light Cycler 480 and related software (Roche Molecular Systems, Inc, Pleasanton, USA).

### Nested PCR amplification of HPV16, 18 and 45 LCR

Using 5 μL of DNA extracted from each of the selected specimen, first round PCRs were performed with the primer sets as indicated in Table 1 for HPV16, 18 and 45. The 50 μL final reaction volume also contained, 400 μM of each dNTP, 1X PCR buffer (200 mM Tris–HCl pH 8.4; 500 mM KCl), 2.5 mM of MgCl_2_, 1.25 μM of each of the two primers, and 5U of AmpliTaq DNA polymerase. The amplifications were achieved with the following conditions; an initial denaturation step at 95 °C for 5 min (10 min for HPV45) was followed by 60 cycles (40 cycles for HPV45) of denaturation at 95 °C for 30 s, primer annealing at 65 °C (55 °C for HPV45) for 30 s and an elongation at 72 °C for 30 s followed with a 7 min final extension at 72 °C and holding the temperature at 4 °C. Extracted DNA form Hela cells, SiHa cells with integrated HPV18, HPV16 genome respectively and confirmed clinical isolate of HPV45 were used as positive control and additional PCR water negative controls were used in each set of amplification.

**Table 1:** List of primer sequences use for first and second round PCR for each og the three HPV genotypes

HPV genotype	First round PCR primers	Second round PCR primers
HPV16	HPV16R1F (5’CACCTACTAATTGTGTTGTGG3’)	HPV16R2F (5’GGGGTACCTCGGTTGCATGCTTTTTGGC3’)
	HPV16R1R (5’GTTTGCACACACCCATG3’)	HPV16R2R (5’GGTCTAGACGGTTTGCACACACCCATGT3’
HPV18	HPV18R1F (5’GTGTTTGTGGTATGGGTGTT3’)	HPV18R2F (5’CGGTTGCATAAACTATGTAT3’)
	HPV18R1R (5’GTATAGTATGTGCTGCCCAA3’)	HPV18R2R (5’TCGGTTGCCTTTGGCTTATG3’),
HPV45 (fragment 1)	HPV45F (5’GACCTCGTAAGCGTCCTGC3’)	HPV45F (5’GACCTCGTAAGCGTCCTGC3’)
	HPV45R (5’GGATGCTGTGTAGTATG CAAGTTTATA3’)	HPV45R (5’GGATGCTGTGTAGTATG CAAGTTTATA3’);
HPV45 (fragment 2)	HPV45F2 (5’GTATGGTGTTACTGTACATA ATTGTGG3’)	HPV45F2 (5’GTATGGTGTTACTGTACATA ATTGTGG3’)
	HPV45R2 (5’CATAGGGTGTGGATACAGTTGTG3’)	HPV45R2 (5’CATAGGGTGTGGATACAGTTGTG3’)
HPV45 (fragment 3)	HPV45F3 5’CTGGCACATTTACAACCCCTAC3’)	HPV45F3 (5’CTGGCACATTTACAACCCCTAC3’)
	HPV45R3 (5’CGCCATCCTGCAATGCAC 3’)	HPV45R3 (5’CGCCATCCTGCAATGCAC 3’)

The second round PCR used 5 μL of the first round product with the second round primer sets indicated in Table 1 for the amplifications. The 100 μL final reaction volume contained 200 μM of each dNTP, 1 X PCR buffer (200 mM Tris–HCl pH 8.4; 500 mM KCl), 2.5 mM of MgCl_2_, 1.25 μM of each of the two primers, and 6.3 U of Taq DNA polymerase. The amplifications were achieved with the same cycling conditions as that of the first round PCR.

### Visualization of second round PCR product

For all the second round PCR products of HPV16, 18 and 45, 20 μL were resolved on a 1.5% agarose gel in 1X TBE buffer with 1 μg/mL ethidium bromide at 120 V for an hour. The gels were documented with GelDoc™ XR+ System (Bio-Rad Laboratory, Mississauga, Canada).

### Sequencing of second round PCR product

The second round PCR products were purified with the Amicon Ultra centrifuge filers (Millipore Ireland Ltd, Tullagreen, Ireland) with slight modification made to the manufactures instruction. Specifically, a filter column was place upright, into a micro-centrifuge tube for each sample, after which 200 μL of PCR water and 80 μL of a PCR product were added. The assembly was spun at 14000xg for 12 min. Additional 100 μL of PCR water were added to each filter column. The filter columns were thereafter transferred, inverted, to a new set of micro-centrifuge tubes and spun for 2 min at 1000xg. The columns were discarded and the eluted DNA in each tube was quantified at 230 nm using NanoDrop ND-100 spectrophotmeter (Thermo Fisher Scientific Inc, Wilmington, USA). The eluted DNA was diluted to 50 ng/μL and sequenced with ABI Applied Biosystems 3730xl DNA Analyzer BigDye® Terminator v 3.0 (Applied Biosystem, Canada). DNA Sequences obtained with the forward and reverse primers by Sanger sequencing were assembled with SeqMan Pro software (DNAStar). The sequences obtained for HPV16, HPV18 and HPV45 PCR products (listed in Additional file 1: Table S1) were aligned to HPV16, HPV18 and HPV45 reference sequences by the Cluster W method, using MEGA 5.2 software. Phylogenetic trees were constructed based on the Test Neighbour Joining using MEGA 6 software.

### Ethical approval and consent to participants

The study that collected the specimen from which the HPV isolates were obtained received ethical approval form the Ethics Review Committee of the Ghana Health Service (ID No GHS-ERC: 06/11/10) and worked within the guidelines of the Ethical Review Committee and conducted the study in accordance to the ethical standards as declared in Declaration of Helsinki and the Belmont Report. Assurances were given and efforts were made to protect the privacy and confidentiality of the participants enrolled and their collected data. As such personal/demographic data as well as data that will be useful for identifying the participants were not included in this study.

## Results

**Fig. 1:**
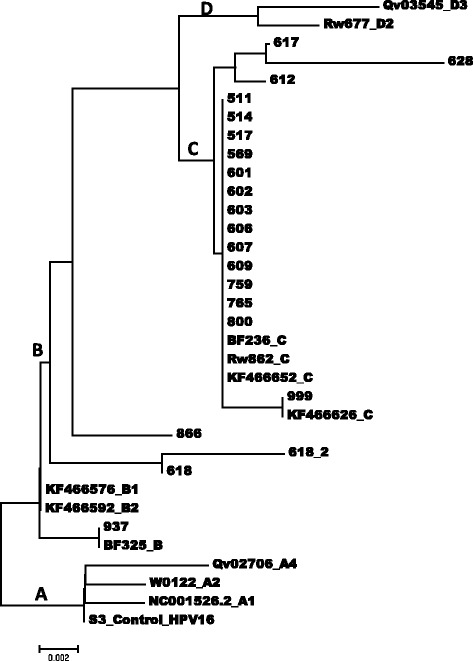
An evolutionary relationship of HPV16 isolates based on sequences between the genome positions 7469 and 7840 within the long control region (LCR.). The evolutionary history was inferred using the Neighbour-Joining method [37]. The optimal tree with the sum of branch length = 0.04170288 is shown. The tree is drawn to scale, with branch lengths in the same units as those of the evolutionary distances used to infer the phylogenetic tree. The evolutionary distances were computed using the Maximum Composite Likelihood method [38] and are in the units of the number of base substitutions per site. The analysis involved 16 nucleotide sequences. Codon positions included were 1^st^ + 2^nd^ + 3^rd ^+ Noncoding. All positions containing gaps and missing data were eliminated. There were a total of 876 positions in the final dataset. Evolutionary analyses were conducted in MEGA 6.0 [39]

**Fig. 2:**
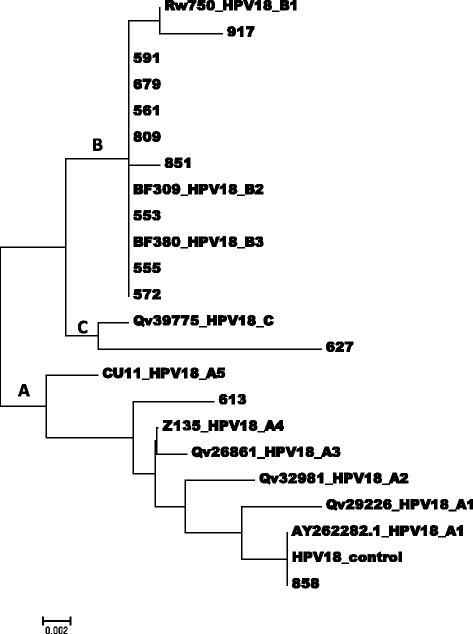
An evolutionary relationship of the taxa of HPV18 isolates based on sequences between the genome positions 7464 and 7839 within the long control region (LCR.). The evolutionary history was inferred using the Neighbor-Joining method [37]. The optimal tree with the sum of branch length = 0.04170288 is shown. The tree is drawn to scale, with branch lengths in the same units as those of the evolutionary distances used to infer the phylogenetic tree. The evolutionary distances were computed using the Maximum Composite Likelihood method [38] and are in the units of the number of base substitutions per site. The analysis involved 16 nucleotide sequences. Codon positions included were 1^st^ + 2^nd^ + 3^rd^ + Noncoding. All positions containing gaps and missing data were eliminated. There were a total of 876 positions in the final dataset. Evolutionary analyses were conducted in MEGA 6.0 [39]

**Fig. 3:**
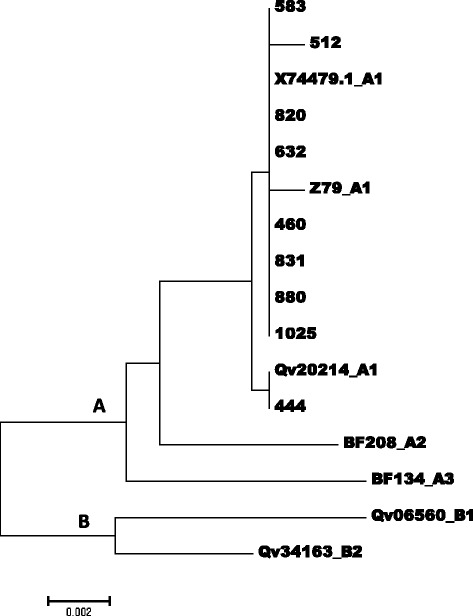
An evolutionary relationship of HPV45 isolates based on sequences between the genome positions 7074 and 7858 within the long control region (LCR.). The evolutionary history was inferred using the Neighbor-Joining method [37]. The optimal tree with the sum of branch length = 0.04170288 is shown. The tree is drawn to scale, with branch lengths in the same units as those of the evolutionary distances used to infer the phylogenetic tree. The evolutionary distances were computed using the Maximum Composite Likelihood method [38] and are in the units of the number of base substitutions per site. The analysis involved 16 nucleotide sequences. Codon positions included were 1^st ^+ 2^nd ^+ 3^rd^ + Noncoding. All positions containing gaps and missing data were eliminated. There were a total of 876 positions in the final dataset. Evolutionary analyses were conducted in MEGA [39]

The obtained partial LCR nucleotide sequences for each of the HPV genotypes were of an average size of 375 bp. These have been deposited in NCBI GenBank and the accession number shown in the Additional file 1: Table S1. The obtained sequences were aligned with the LCR positions of the complete genome of the HPV16 prototype NC0015626.2 [33, 34], the HPV18 prototype AY262282_A1 [35] and the HPV45 prototype X74479.1 [36]. The alignments also included selected HPV variants with very similar sequences obtained by an NCBI BLAST of the isolates’ sequences. A neighbour joining phylogenetic tree was constructed for each HPV type (Figs. 1–3). All 20 HPV16 specimen were confirmed by positive results for the LCR HPV16 type specific PCR. Nucleotide sequence variations were observed at 30 positions within the LCR (Table 2). Of these 30 positions, 19 were the known variations often observed for African variants, while the other 11 positions had variations unique to the HPV16 isolates of this study (Table 2). The phylogenetic tree showed that about 85% of the HPV16 isolates were of the lineage C, 5% of lineage B, both of which were previously known as African variants (Fig. 1) and the lineages of two isolates (10%), isolate-866 and isolate 618 were not clearly identified, although they were most likely to belong to lineage B.

**Table 2:**
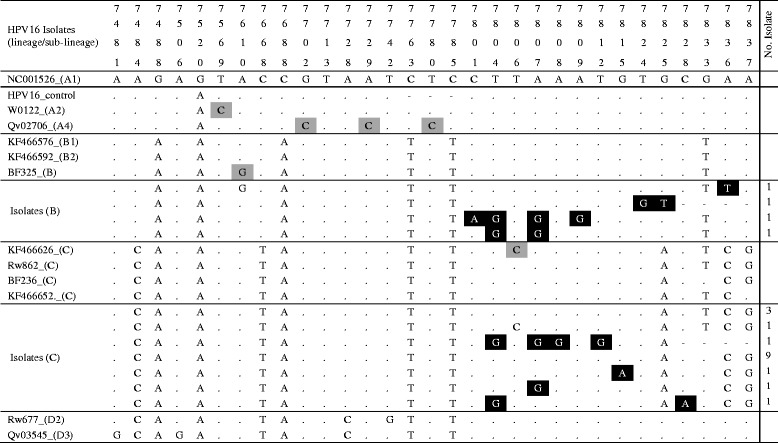
Sequence variations within nucleotide positions 7469 to 7840 (LCR) of HPV16 variants

Each column and the numbers in first four rows represents a single specific nucleotide position in the LCR. The fifth row shows the nucleotide (indicated by the standard letter of its nitrogenous bases; (A) adenine, (C) cytosine, (G) guanine, and (T) thymine) at each specific position for the LCR of the prototype HPV16 isolates, NC001526. The sixth row is the sequence of the SiHa positive control. Alphanumeric in column are NCBI BLAST obtained isolates of known lineage. The following were the indication:  variation unique to isolates of this study,  variations that differentiate known HPV16 lineages,  same nucleotide as prototype isolate,  deletion of nucleotide at that position. Isolates’ GenBank accession numbers are shown in Additional file 1: Table S1

Of the 15 specimen positive for HPV18 isolates, 13 were confirmed as positive by the LCR HPV18 type specific PCR, one of which gave poor sequence data. Nucleotide variations were determined in 35 positions, 22 of which were known variations of the lineages B and C (Africa variants) of HPV 18. The other 13 variations were unique to the isolates of this study (Table 3). The phylogenetic tree (Fig. 2) showed that most of the HPV18 isolate were of the lineages B2 (6 of 12), B3 (2 of 12), B1 (1 of 12) and C (1 of 12) (African variants) and only two isolates (isolates 613 and isolate 858) were of the non-African lineage (lineage A). Of the 18 specimen confirmed as HPV45 genotypes, 13 gave amplification products and 9 has good sequences. (Table 4). Compared to the HPV45 prototype, nucleotide sequence variations were determined in 35 positions, 2 of which were unique to the sequences of 8 isolates of this study. The phylogenetic tree showed that all the HPV45 were of the lineage A1 (Fig. 3).

**Table 3:**
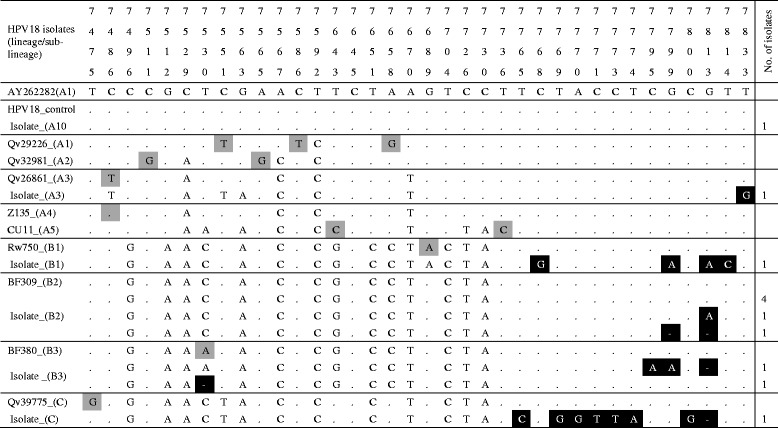
Sequence variations within nucleotide positions 7464 and 7839 (LCR) of HPV18 variants

Each column and the numbers in first four rows represents a single specific nucleotide position in the LCR.. The fifth row shows the nucleotide (indicated by the standard letter of its nitrogenous bases; (A) adenine, (C) cytosine, (G) guanine, and (T) thymine) at each specific position for the LCR of the prototype HPV18 isolates, AY262282. The sixth row is the sequence of the HaLa positive control. Alphanumeric in column are NCBI BLAST obtained isolates of known lineage. The following were the indication:  variation unique to isolates of this study,  variations that differentiate known HPV18 lineages,  same nucleotide as prototype isolate,  deletion of nucleotide at that position. Isolates’ GenBank accession numbers are shown in Additional file 1: Table S1

**Table 4:**
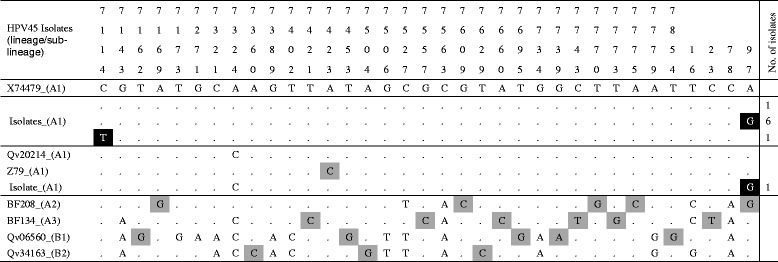
Nucleotide sequence variations between positions 7074 and 7858 (LCR) of HPV45 variants

Each column and the numbers in first four rows represents a single specific nucleotide position in the LCR.. The fifth row shows the nucleotide (indicated by the standard letter of its nitrogenous bases; (A) adenine, (C) cytosine, (G) guanine, and (T) thymine) at each specific position for the LCR of the prototype HPV45 isolates, X74479. The sixth row is the sequence of the HaLa positive control. Alphanumeric in column are NCBI BLAST obtained isolates of known lineage. The following were the indication:  variation unique to isolates of this study,  variations that differentiate known HPV45 lineages,  same nucleotide as prototype isolate,  deletion of nucleotide at that position

## Discussion

This long control region of the HPV genome has been shown to be very informative and therefore very useful in the study of HPV variants [7, 12–14], as such it was used in this study. A close look at the phylogenetic tree (Fig. 1) suggests that Isolate-937, isolate-866 and isolate-618, may to belong to the HPV16 lineage B (Africa type 1 variants), although it showed that isolates-866, isolate-618 and 618_2 clustered slightly differently. A complete genome sequencing, which was not within the reach of this study would have clarified the lineages of these isolates [5]. Although not clearly understood, repeated PCR and subsequent sequencing of a single specimen produced the isolate-618 and isolate-618_2 with varied branch length and at two positions (Fig. 1). More importantly, the phylogenetic tree showed that the length of sequence obtained in this study were not enough to separate the sub-lineages of the HPV16 lineage B, since the nucleotide positions obtained in this study (Table 2) did not include the position (7438) which distinguishes the sub-lineages B1 and B2 (Af-1a and Af-1b respectively). This was evident by the close clustering of the previously described HPV16 sub-lineage B1 (KF466576) and HPV16 sub-lineage B2 (KF466592) in the phylogenetic tree (Fig. 1). These two variants were distinguished from each other by the substitution A7438C in an earlier study [7]. Therefore, this may imply that the clustering of isolates 866 and 618 may have been different with a longer sequence.

A more detail investigation of the variations in the sequences (Table 2) showed how very similar these isolates were and the bases for the differences seen in the phylogenetic tree. It also showed nucleotide variations unique to the isolates of this study. The occurrence of most of these unique variability specific to the isolates of this study, occurred between the genome positions 7801 and 7828. These may have implication for transcription activities in pathogenic pathways and therefore in oncogenesis, since this range of positions include the binding sites for transcription factors, activator protein 1 (AP1) and Octamer-binding protein 1 [13] which are involved the expression of the genome of the HPV.

The phylogenetic tree presented as Fig. 2 indicates that the sub-lineages B1 and B2 (African sub-lineage 1, old nomenclature) of the HPV18 isolates were the commonest. The sub-lineage B3 and lineage C (African sub-lineage 2, old nomenclature) were also common. Interestingly, only a few were of the A1, A2, and A3 sub-lineages (European sub-lineage (Eur), old nomenclature). A detailed comparison with sequences obtained by an NCBI BLAST and the HPV18 prototype AY262282_A1 [35] showed variations that were unique to the sequence of the isolates of this study, in addition to variations well known for the identified lineages. In all, 6 of the 11 isolates identified in this study had not been described by other studies while the other have been [6, 15]. Most of the nucleotide variations specific to isolates of this study were located within the genome positions 7769 to 7714 and 7795 to 7814; regions where the binding sites for the transcription factors NF1, YY1 and AP1 are located. These changes may have implication for transcription activities in pathogenic pathways [13] and therefore the oncogenic potential of the isolates of this study at the level of sub-lineages. However, it must be noted that these HPVs were isolated from women with normal cytology results. Therefore, a follow-up study may provide more information relating to the potential relationship between these sequence variations and oncogenicity.

All the 9 HPV45 genotypes sequenced were identified without difficulty and were determined to belong to the HPV45 lineage A1 (Table 4). Although the lineages of most of the isolates of this study were identified much clearly, the primary limitation of this study was that in the absence of a complete genome sequence, or the sequence of other genes, such as the E6 and E7 gene, the lineages of some isolate were not clearly identified and the few isolates that did not cluster within known lineage cannot be said to indicate new lineages.

## Conclusion

This study provides the first DNA sequence data on the variants/lineages of HPV 16, 18 and 45 isolates from Ghana and has shown that predominately, these genotypes are of the Africa lineages. The observance of specific sequence variation within region associated with transcription binding, indicates a potential for differential oncogenicity of the isolate. The fact that these were obtained from the specimen of women with normal cytology, these data will be useful for comparison with variants of these genotypes from women with the different form of ICC and different grades of cervical lesions to improve our understanding of relationship between lineage and oncogenicity. However, there is need for additional gene or a full genome sequencing to better clarify the classification of a few of the isolates that did not cluster with most of the isolates within a clear lineage.

## Additional file

Additional file 1: Table S1. Isolates’ GenBank accession numbers and lineage classifications. (DOCX 12 kb)

## Abbreviations

ADC: Adenocarcinoma
ADSC: Adenosquamous carcinoma
EAS: European-Asian
HPV: Human Papillomavirus
ICC: Invasive cervical cancer
LCR: Long control region
SCC: Squamous cell carcinoma
