# Supplementary material for: Unique LCR variations among lineages of HPV16, 18 and 45 isolates from women with normal cervical cytology in Ghana
Source: Virol J. 2017 Apr 21;14:85. doi: 10.1186/s12985-017-0755-z (PMC5401561; doi:10.1186/s12985-017-0755-z)
Supplement: Additional file 1: Table S1. — Isolates’ GenBank accession numbers and lineage classifications. (DOCX 12 kb) [file 12985_2017_755_MOESM1_ESM.docx]

**Table S1: Isolates’ GenBank accession numbers and lineage classifications**

| Isolate | GenBank Accession number | Genotype | Lineage |
| --- | --- | --- | --- |
| 999 | KM226842.1 | HPV16 | C |
| 937 | KM226843.1 | HPV16 | B |
| 866 | KM226844.1 | HPV16 | B |
| 800 | KM226845.1 | HPV16 | C |
| 765 | KM226846.1 | HPV16 | C |
| 759 | KM226847.1 | HPV16 | C |
| 628 | KM226848.1 | HPV16 | C |
| 618_2 | KM226849.1 | HPV16 | B |
| 618 | KM226850.1 | HPV16 | B |
| 617 | KM226851.1 | HPV16 | C |
| 612 | KM226852.1 | HPV16 | C |
| 609 | KM226853.1 | HPV16 | C |
| 607 | KM226854.1 | HPV16 | C |
| 606 | KM226855.1 | HPV16 | C |
| 603 | KM226856.1 | HPV16 | C |
| 602 | KM226857.1 | HPV16 | C |
| 601 | KM226858.1 | HPV16 | C |
| 569 | KM226859.1 | HPV16 | C |
| 517 | KM226860.1 | HPV16 | C |
| 514 | KM226861.1 | HPV16 | C |
| 511 | KM226862.1 | HPV16 | C |
| 561 | KM226863.1 | HPV18 | B |
| 591 | KM226864.1 | HPV18 | B |
| 627 | KM226865.1 | HPV18 | C |
| 679 | KM226866.1 | HPV18 | B |
| 851 | KM226867.1 | HPV18 | B |
| 553 | KM226868.1 | HPV18 | B |
| 555 | KM226869.1 | HPV18 | B |
| 572 | KM226870.1 | HPV18 | B |
| 613 | KM226871.1 | HPV18 | A |
| 809 | KM226872.1 | HPV18 | B |
| 858 | KM226873.1 | HPV18 | A |
| 917 | KM226874.1 | HPV18 | B |
